# Mitochondrial Structural Changes in the Pathogenesis of Diabetic Retinopathy

**DOI:** 10.3390/jcm8091363

**Published:** 2019-09-01

**Authors:** Sayon Roy, Dongjoon Kim, Aravind Sankaramoorthy

**Affiliations:** 1 Department of Medicine, Boston University School of Medicine, Boston, MA 02118, USA; 2 Department of Ophthalmology, Boston University School of Medicine, Boston, MA 02118, USA

**Keywords:** mitochondrial morphology, diabetes, retina, diabetic retinopathy

## Abstract

At the core of proper mitochondrial functionality is the maintenance of its structure and morphology. Physical changes in mitochondrial structure alter metabolic pathways inside mitochondria, affect mitochondrial turnover, disturb mitochondrial dynamics, and promote mitochondrial fragmentation, ultimately triggering apoptosis. In high glucose condition, increased mitochondrial fragmentation contributes to apoptotic death in retinal vascular and Müller cells. Although alterations in mitochondrial morphology have been detected in several diabetic tissues, it remains to be established in the vascular cells of the diabetic retina. From a mechanistic standpoint, our current work supports the notion that increased expression of fission genes and decreased expression of fusion genes are involved in promoting excessive mitochondrial fragmentation. While mechanistic insights are only beginning to reveal how high glucose alters mitochondrial morphology, the consequences are clearly seen as release of cytochrome c from fragmented mitochondria triggers apoptosis. Current findings raise the prospect of targeting excessive mitochondrial fragmentation as a potential therapeutic strategy for treatment of diabetic retinopathy. While biochemical and epigenetic changes have been reported to be associated with mitochondrial dysfunction, this review focuses on alterations in mitochondrial morphology, and their impact on mitochondrial function and pathogenesis of diabetic retinopathy.

## 1. Introduction

Time-lapse videography of live cells has documented spectacular mitochondrial movement within a cell where each mitochondrion movement occurs in the direction of its longitudinal axis. The speed at which this movement takes place can vary substantially from 2 to 30 µm/min along the direction of its long axis. Long gone are the days when mitochondria were believed to be static organelles. In fact, movement among mitochondria facilitates connectivity between mitochondria resulting in a dynamic mitochondrial network. Subtle changes in mitochondrial shape seen as local narrowing/contractions and thickening/relaxations reflect a “worm-like swimming” movement coupled with a remarkable phenomenon of fission and fusion, orchestrating the dynamic nature of the mitochondrial network. These changes in mitochondrial structure are important to the normal function of cells. Indeed, mitochondrial shape change can rapidly flip from facilitating normal cell function to promoting apoptotic cell death. The ability of high glucose (HG) condition to promote mitochondrial fragmentation has captured the interest of several labs including ours. Studies from our lab have shown that disturbances to the intrinsic dynamic changes in mitochondrial network can negatively impact cell survival. In HG condition, mitochondrial fragmentation led to compromised metabolic changes, ultimately promoting apoptosis [1,2,3].

## 2. Consequences of Mitochondrial Dysfunction in Microangiopathy

To maintain proper functionality, the mitochondrial network undergoes constant dynamic reorganization through fission and fusion. In diabetes, mitochondrial dysfunction is attributable, at least in part, to altered mitochondrial morphology, specifically mitochondrial fragmentation. Changes in mitochondrial dynamics have been implicated in diabetic retinopathy [1,3], nephropathy [4,5], and neuropathy [6]. Table 1 shows a list of mitochondrial morphology changes in various tissues affected by diabetes [1,2,3,7,8,9,10,11,12,13,14,15,16,17,18,19,20].

**Figure 1:**
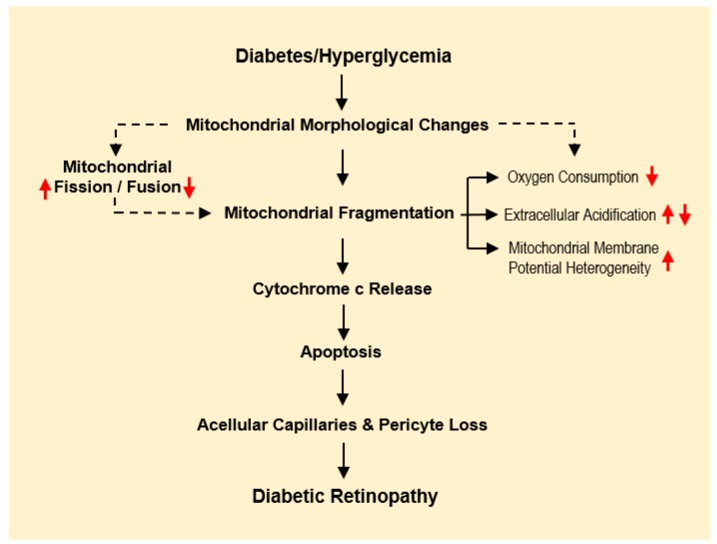
Changes in mitochondrial morphology and its implications in diabetic retinopathy. Diagram illustrates the effects of diabetes/hyperglycemia on mitochondrial morphology changes leading to mitochondrial dysfunction, contributing to the development and progression of diabetic retinopathy. Dashed arrows suggest possible mechanisms that are still under investigation.

Although evidence suggests that HG-induced changes in mitochondrial structure contribute to cellular dysfunction, the exact molecular mechanism(s) underlying these changes are still under intense investigation. Altered mitochondrial dynamics negatively impact mitochondrial respiration, membrane potential, induce cytochrome c release, and promote apoptosis [24,25,26,27]. Taken together, current findings indicate that HG-induced mitochondrial morphological changes play a key role in the development of retinal lesions associated with diabetic retinopathy (Figure 1), as well as diabetic microangiopathy in general.

## 3. Changes in Mitochondrial Morphology in Diabetic Retinopathy

Mitochondrial morphological changes have been associated with alterations in normal cellular homeostasis such as energy production [28], calcium production, mitochondrial DNA distribution, apoptosis, and mitophagy [29]. The fine balance between fission and fusion dictates normal cellular homeostasis. Studies have shown that ultrastructural changes in the shape and size of mitochondria is linked to the metabolic state of the cell. Studies examining the relationship between mitochondrial morphology and cellular metabolic states indicate mitochondrial size to be smaller in energetically inactive or quiescent cells than in energetically active cells [30,31].

Changes in mitochondrial morphology such as increased mitochondrial fragmentation have been documented in coronary endothelial cells in diabetic animal models [14]. Similarly, reduced mitochondrial size in skeletal muscle fiber [13,32], and mitochondrial swelling in hepatocytes [33] have been identified in patients with type 2 diabetes [13,32,33]. Alteration in mitochondrial morphology and its impact on retinal function has gained significant attention in studies of the pathogenesis of diabetic retinopathy; clinical trials are currently underway investigating the effects of diabetes and hyperglycemia on mitochondrial fragmentation.

## 4. Alterations in Mitochondrial Structure: Fission, Fusion, and Fragmentation

Mitochondria are dynamic organelles which undergo constant modifications in their shape and size. This process may vary among different cell types. The maintenance of mitochondrial structure involves a delicate balance between fission and fusion events [34]. The fission and fusion machinery operates continuously, resulting in upkeep of the dynamic mitochondrial network. Hence, fission and fusion cycles are necessary for mitochondrial growth, mitochondrial redistribution, and maintenance of mitochondrial length and shape [34]. In general, mitochondrial fusion positively impacts the maintenance of mitochondrial structure, whereas excessive mitochondrial fission is seen as deleterious in disease processes [35]. Dynamin family member proteins, which serve as GTPases, are primarily involved in the regulation of fission and fusion events [34]. Drp1 and Fis1 regulate mitochondrial fission, while fusion involving mitochondrial outer membranes is mediated by membrane-anchored dynamin family members Mfn1 and Mfn2, and fusion in the mitochondrial inner membrane is regulated by a single dynamin member Opa1 [35,36,37]. When mitochondria are partially damaged due to metabolic or oxidative stress, the mitochondria attempts a repair process wherein parts of the damaged mitochondria are combined by complementary fusion [35]. While mitochondrial fission is a necessary process for the maintenance of mitochondrial network, for mitochondrial biogenesis and the recycling of dysfunctional, damaged mitochondria, excessive fission can trigger mitochondrial fragmentation and apoptosis [35]. Both mitochondrial fission and fusion are indispensable events for proper cellular functions [38]. As such, disruption in these processes can have profound effects and contribute to diseases such as diabetes and diabetic retinopathy [6,12,18,21,22,23,39,40,41,42,43,44,45].

Increasing evidence points to abnormal mitochondrial structural changes as being one of the pathophysiological processes involving mitochondrial dysfunction in diabetic retinopathy. Current understanding suggests that alteration in fusion and fission gene expression may negatively impact mitochondrial structure. Taken together, these findings suggest that high glucose-induced mitochondrial structural damage involving excessive mitochondrial fragmentation plays a critical role in promoting apoptotic cell death associated with diabetic retinopathy.

## 5. HG-Induced Altered Mitochondrial Function Compromises Cellular Respiration

Oxygen consumption rate is a widely used index for studying mitochondrial metabolic activity [19,20,46]. In addition, extracellular acidification rate is useful in determining glycolytic flux [47]. Studies have shown that HG negatively impacts cellular respiration in retinal vascular cells. Reduced oxygen consumption is accompanied by altered mitochondrial membrane potential heterogeneity, cytochrome c release, and increased apoptosis [1]. Decreased oxygen consumption rate has been reported in isolated mitochondria of diabetic rat retinas concomitant with decreased CO_2_ production. In early stages of diabetes, retinal vascular cells exhibit an adaptive response whereas reduced cellular respiration occurs in late stages of diabetes [48].

Retinal endothelial cells grown in high glucose condition exhibit mitochondrial fragmentation concomitant with altered membrane potential heterogeneity [1]. Importantly, metabolic analysis revealed increased extracellular acidification, but reduced steady state/maximal oxygen consumption rate [1]. Increased extracellular acidification rate in these cells may be attributable to a compensatory mechanism counterbalancing high glucose-induced decreased mitochondrial oxygen consumption. Functionally, these respiratory changes are associated with increased cytochrome c release and apoptosis, suggesting that alteration in mitochondrial metabolism could be deleterious and promote cell loss seen in diabetic retinopathy.

In retinal pericytes, similar changes were observed under high glucose conditions. Excessive mitochondrial fragmentation and increased membrane potential heterogeneity were noted in retinal pericytes grown under high glucose medium [2]. While changes in membrane potential heterogeneity and steady state and maximum oxygen consumption rate were similar to those of retinal endothelial cells, extracellular acidification rate was decreased in pericytes under high glucose condition compared to those of endothelial cells [2]. This discrepancy may be due to the inability to compensate for high glucose-induced reduction in mitochondrial oxygen consumption in retinal pericytes. The observed difference in metabolic activity between endothelial cells and pericytes may be explained by differential transport of glucose between the two cell types. Specifically, GLUT1 activity was found to be reduced in pericytes, but not in endothelial cells under high glucose conditions [49]. Therefore, there may be a disparity in extracellular acidification and possibly even glycolytic levels in retinal pericytes and endothelial cells in response to high glucose. As expected, these metabolic changes in the mitochondria induced by high glucose led to increased apoptotic cell death in retinal pericytes. Further, this suggests that both retinal endothelial cells and pericytes may be susceptible to mitochondrial metabolic dysfunction induced by high glucose.

While mitochondrial dysfunction has been documented in retinal vascular cells, limited information is available with respect to retinal glial cells under high glucose conditions. Our recent study elucidated that retinal Müller cells grown in high glucose conditions also exhibit mitochondrial fragmentation with concomitant increase in membrane potential heterogeneity [3]. Comparable to retinal pericytes, retinal Müller cells showed decreased oxygen consumption rate. Though unlike retinal endothelial cells, retinal Müller cells showed decreased extracellular acidification rate, concomitant with cytochrome c release and apoptosis [3]. Taken together, these findings provide clear evidence that high glucose contributes to mitochondrial dysfunction in retinal vascular and glial cells by compromising mitochondrial function and cellular metabolism, thereby promoting apoptosis associated with the development of acellular capillaries, pericyte loss, and neuronal injury in diabetic retinopathy.

## 6. Mitochondrial Dysfunction-Mediated Apoptosis in Diabetic Retinopathy

**Figure 2:**
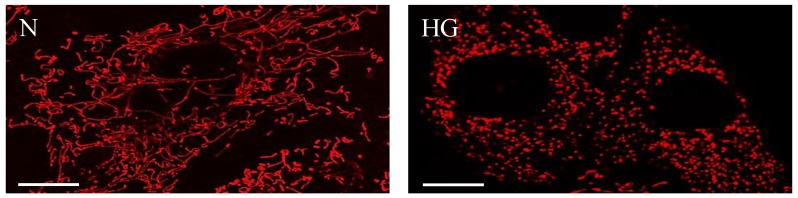
High glucose compromises mitochondrial morphology and promotes mitochondrial fragmentation. Representative confocal images of RRECs grown in normal (N) medium (**left**) stained with mitotracker red show long, tubular networks of mitochondria. In parallel, cells grown in high glucose (HG) medium (**right**) exhibit significant mitochondrial fragmentation. Scale bars: 10 µm.

Apoptosis or programmed cell death was initially believed to be regulated by the nucleus until it was discovered that enucleated cells also undergo apoptosis, and that the intrinsic apoptotic process is regulated by mitochondria [50]. A study showed that Bcl-2 is localized in the inner mitochondrial membrane [51], where it can perform either anti- or pro-apoptotic actions, thus confirming mitochondria’s ability to regulate apoptosis. Furthermore, opening and closure of mitochondria membrane transition pores [52] and subsequent release of cytochrome c [53] is known to modulate the intrinsic apoptotic pathway. While apoptosis can be triggered through various mechanisms, cytochrome c release is a pivotal culminating “point of no return” for the commitment towards apoptosis. Studies have shown that cytochrome c release can develop as a result of altered mitochondrial dynamics [1], activation of Drp1, a fission protein [54], and mitochondrial fragmentation. Our studies with retinal vascular cells have shown that HG-induced mitochondrial fragmentation (Figure 2) concomitant with altered membrane potential heterogeneity, reduced oxygen consumption rate, increased extracellular acidification, and that cytochrome c release promotes apoptosis, suggesting that mitochondrial dysfunction involving structural changes can mediate the retinal vascular cell death seen in diabetic retinopathy [1,2].

## 7. Mitochondrial Cx43 (mtCx43) Abnormalities and Mitochondrial Fragmentation

Presence of Cx43 in the mitochondria is a relatively recent discovery and has gained significant attention [55,56,57,58,59,60,61,62,63,64,65,66,67,68,69,70,71]. A study showed that inhibition of mtCx43 activity can promote cytochrome c, and thereby promote apoptosis [60]. In our lab using confocal microscopy, green fluorescent protein-tagged Cx43 was identified mostly in the inner mitochondrial membrane of retinal endothelial cells. Following Western blot analysis we also demonstrated that mtCx43 expression levels are significantly downregulated under high glucose condition, which in turn triggers mitochondrial fragmentation and subsequent release of cytochrome c in retinal endothelial cells [70]. These findings suggest that functional activity of mtCx43 channels is critical for maintaining mitochondrial morphology. Interestingly, inhibition of mtCx43 activity in isolated mitochondria promoted cytochrome c release [70], suggesting that mtCx43 changes may contribute to overall mitochondrial dysfunction evident in retinal vascular cells associated with diabetic retinopathy. While the exact role of mtCx43 in retinal vascular cells is not well understood, the scientific literature suggests that mtCx43 activity is mostly beneficial and that it confers cytoprotective effects in cardiac cells through diminishing reactive oxygen species (ROS) production in the cytosol and mitochondria, reducing mitochondrial calcium overload, mitochondrial membrane depolarization, and preventing the release of cytochrome c [65,67,68]. In addition, inhibition of mtCx43 has been shown to promote ROS release and induction of autophagy, indicating that proper mtCx43 activity is necessary to maintain mitochondrial integrity and metabolic activity of brown adipose tissue [61]. Collectively, these findings indicate that HG-induced downregulation of mtCx43 protein contributes to decreased Cx43 channel activity, altered mitochondrial morphology, and cytochrome c release. This could provide a framework for a novel mechanism underlying apoptotic retinal vascular cell death in diabetic retinopathy.

## 8. Strategies Inhibiting Mitochondrial Fragmentation and Dysfunction

It is well established that mitochondrial fragmentation disrupts cellular homeostasis through destabilization of mitochondrial membranes, ultimately leading to cytochrome c release, apoptotic cell death, and contributing to mitochondria-related disorders. Excessive mitochondrial fission and altered mitochondrial dynamics have been documented to play a role in several disease processes including cardiovascular disease, neurodegenerative disorders [72], Huntington’s disease, diabetes, mitochondrial diseases, Alzheimer’s disease, Parkinson’s disease, multiple sclerosis, and amyotrophic lateral sclerosis [73]. Involvement of increased levels of fission gene expression, in particular, Drp1, appears to play a central role in these disease processes. While studies are underway examining molecules capable of inhibiting excessive mitochondrial fragmentation for treatment of patients with mitochondria-related disorders, strategies to combat mitochondrial fragmentation using drugs promoting mitochondrial fusion are also underway. Recent research has revealed that abnormal mitochondrial dynamics is an early event in diabetes. Although mitochondrial fusion and fission are necessary events for maintenance of functional mitochondria and the dynamic mitochondrial network, the balance between fission and fusion is a delicate process. Excessive fission and reduced fusion can lead to accumulation of mitochondrial fragments, leading to mitochondrial dysfunction and development of mitochondria-related disease processes. In diabetes, strategies are being tested for preventing mitochondrial fragmentation that includes administration of mitochondrial division inhibitor-1 (mdivi-1), dynasore, P110, and 15-oxospiramilactone [42]. A clinical trial investigating mitochondrial dynamics has begun, looking into effects of dietary stearic acid on mitochondrial fusion [74]. Another clinical trial currently ongoing entitled “Hyperglycemia and Mitochondrial Function in The Endothelium of Humans” (ClinicalTrials.gov Identifier #NCT02682342) is examining the effects of hyperglycemia on mitochondrial network, specifically fragmentation and fission in diabetic patients. The future prospect of targeting excessive mitochondrial fragmentation as a therapeutic strategy for diabetic retinopathy is promising.

## 9. Conclusions

Recent strategies for preventing excessive mitochondrial fragmentation in diseases have gained attention. It is evident that mitochondrial morphology changes play an important role in contributing to biochemical and molecular alterations underlying disease processes, including those of diabetic retinopathy. While mitochondrial fragmentation has been implicated in pathophysiological changes involving kidneys, liver, heart, pancreas, and skeletal muscles in diabetes, only limited information is available with respect to diabetic retinopathy. The consequences of mitochondrial fragmentation are significant, affecting various functions including cellular metabolism, cellular respiration, and triggering apoptotic death. Each of these cellular events is closely associated with the development and progression of retinal lesions in diabetic retinopathy. As such, an understanding of molecular mechanisms underlying mitochondrial structural changes is necessary. The challenge would be to identify novel targets for maintaining intact mitochondrial morphology in diabetic retinopathy.

## Figures and Tables

**Table 1:** Mitochondrial morphology changes in diabetic tissues.

Tissue Type	Cell Type	Species	Changes in Mitochondrial Morphology	References
Retina	Retinal endothelial cells	Rat	Fragmentation	[1]
	Retinal pericytes	Bovine	Fragmentation	[2]
	Retinal Müller cells	Rat	Fragmentation	[3]
	Retinal Müller cells	Human	Fragmentation	[21]
	Retinal pigmented epithelial cells	Human	Fragmentation	[22]
Kidney	Renal glomerular cells	Human	Fragmentation	[7]
	Proximal tubule epithelial cells	Rat	Fragmentation	[9]
	Proximal tubule epithelial cells	Human	Fragmentation	[23]
	Podocytes	Mouse	Fragmentation	[16]
Liver	Hepatocytes	Rat	Increased mitochondria size and density	[11]
	Epithelial cells	Rat	Fragmentation	[19,20]
Heart	Coronary endothelial cells	Mouse	Fragmentation	[14]
	Myoblasts	Rat	Fragmentation	[19]
	Ventricular myocytes	Rat	Fragmentation	[20]
	Aortic endothelial cells	Bovine	Fragmentation	[20]
	Aortic smooth muscle cells	Mouse	Fragmentation	[20]
	Aortic endothelial cells	Human	Fragmentation	[18]
	Venous endothelial cells	Human	Fragmentation	[18]
Pancreas	β-islet cells	Rat	Fragmentation	[10]
	β-islet cells	Mouse	Fragmentation	[15]
Fat	Adipocytes	Mouse	Decreased mitochondria size	[8]
Skeletal Muscle	Myoblast	Mouse	Fragmentation	[12]
	Skeletal muscle cells	Human	Decreased mitochondria size and larger vacuoles	[13]
	Skeletal muscle cells	Mouse	Swelling and lysis of mitochondrial cristae	[17]
